# Bis{1-[(4-methyl­phen­yl)imino­meth­yl]-2-naphtho­lato-κ^2^ *N*,*O*}copper(II)

**DOI:** 10.1107/S1600536810030667

**Published:** 2010-08-11

**Authors:** Peihua Zhu, Hongyan Wang, Yan Wang, Yanli Chen, Qin Wei

**Affiliations:** a School of Chemistry and Chemical Engineering, University of Jinan, Jinan 250022, People’s Republic of China;

## Abstract

In the title complex, [Cu(C_18_H_14_NO)_2_], the Cu^II^ ion lies on an inversion center and is coordinated in a slightly distorted square-planar environment. The 1-[(4-methyl­phen­yl)imino­meth­yl]-2-naphtho­late ligands are coordinated in a *trans* arrangement with respect to the N and O atoms.

## Related literature

For background information and applications of Schiff base complexes, see: Adsule *et al.* (2006 ▶); Barton *et al.* (1979 ▶); Cohen *et al.* (1964 ▶); Henrici-Olive & Olive (1984 ▶); Erxleben & Schumacher (2001 ▶). For related structures, see: Kani *et al.* (1998 ▶); Lo *et al.* (1997 ▶); Ünver (2002 ▶).
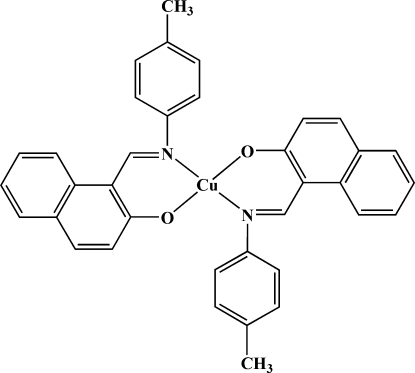


## Experimental

### 

#### Crystal data

[Cu(C_18_H_14_NO)_2_]
*M* *_r_* = 584.14
Triclinic, 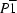

*a* = 7.0948 (6) Å
*b* = 10.2335 (7) Å
*c* = 10.5784 (10) Å
α = 104.559 (7)°
β = 98.728 (7)°
γ = 102.573 (7)°
*V* = 708.01 (10) Å^3^
*Z* = 1
Mo *K*α radiation
μ = 0.81 mm^−1^
*T* = 293 K
0.25 × 0.12 × 0.11 mm

#### Data collection

Bruker APEXII CCD area-detector diffractometer
Absorption correction: multi-scan (*SADABS*; Sheldrick, 2003 ▶) *T*_min_ = 0.824, *T*_max_ = 0.916
7213 measured reflections
2878 independent reflections
2395 reflections with *I* > 2σ(*I*)
*R*_int_ = 0.028

#### Refinement

*R*[*F* ^2^ > 2σ(*F* ^2^)] = 0.032
*wR*(*F* ^2^) = 0.076
*S* = 1.01
2878 reflections
188 parameters
H-atom parameters constrained
Δρ_max_ = 0.24 e Å^−3^
Δρ_min_ = −0.18 e Å^−3^

### 

Data collection: *APEX2* (Bruker, 2004 ▶); cell refinement: *SAINT-Plus* (Bruker, 2001 ▶); data reduction: *SAINT-Plus*; program(s) used to solve structure: *SHELXS97* (Sheldrick, 2008 ▶); program(s) used to refine structure: *SHELXL97* (Sheldrick, 2008 ▶); molecular graphics: *SHELXTL* (Sheldrick, 2008 ▶); software used to prepare material for publication: *SHELXTL*.

## Supplementary Material

Crystal structure: contains datablocks global, I. DOI: 10.1107/S1600536810030667/lh5095sup1.cif

Structure factors: contains datablocks I. DOI: 10.1107/S1600536810030667/lh5095Isup2.hkl

Additional supplementary materials:  crystallographic information; 3D view; checkCIF report

## Figures and Tables

**Table:** 

Cu1—O	1.8837 (12)
Cu1—N	1.9848 (14)

**Table:** 

O^i^—Cu1—O	180
O^i^—Cu1—N	89.58 (5)
O—Cu1—N	90.42 (5)
N—Cu1—N^i^	180

Symmetry code: (i) 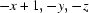.

**Fig. 1.**
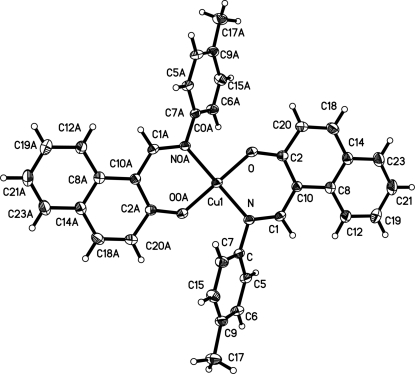
The molecular structure, with atom labels and 25% probability displacement ellipsoids for non-H atoms (symmetry code: (A) -x+1, -y, -z).

## supplementary crystallographic information

### Comment

Schiff bases and their metal complexes have aroused considerable attention, mainly because of their interesting structures and potential applications, *e.g.* catalytic activity (Henrici-Olive & Olive *et al.*, 1984), photochromic properties (Cohen *et al.*, 1964), biological activity (Barton *et al.*, 1979). Additionally, copper (II) complexes of Schiff bases have been reported for their applications in the design and construction of new magnetic materials (Erxleben & Schumacher, 2001), and their cellular proteasome activity (Adsule *et al.*, 2006). Herein we report the synthesis and crystal structure of the title complex.

The molecular structure of the title complex is shown in Fig. 1. The Cu^II^ ion is coordinated by two O atoms and two N atoms of two bidentate schiff base ligands to form a square-planar geometry in a *trans* arrangement. The Cu—N and Cu—O bond lengths agree with those in related complexes (e.g. Kani *et al.*, 1998; Lo *et al.*, 1997; Ünver, 2002).

### Experimental

Copper(II) acetate hydrate (0.199 g, 0.001 mol) in methanol (50 ml) and *N*-(*p*-Tolyl)-2-hydroxy-1-naphthaldimine (0.586 g, 0.002 mol) in acetonitrile(75 ml) were mixed and heated at 333 K for 1 h. The solution was filtered and the filtrate kept in a beaker at room temperature for crystallization. Black crystals started appearing after 3 days and were then collected, 0.621 g (79%) yields.

### Refinement

Hydrogen atoms were placed in calculated positions and refined using a riding-model approximation with C—H = 0.93 Å, *U*_iso_ = 1.2*U*_eq_ (C) for aromatic H atoms and C—H = 0.96 Å, *U*_iso_ = 1.5*U*_eq_ (C) for methyl H atoms.

### Crystal data

**Table:**

[Cu(C_18_H_14_NO)_2_]	*Z* = 1
*M**_r_* = 584.14	*F*(000) = 303
Triclinic, *P*1	*D*_x_ = 1.370 Mg m^−^^3^
Hall symbol: -P 1	Mo *K*α radiation, λ = 0.71073 Å
*a* = 7.0948 (6) Å	Cell parameters from 1252 reflections
*b* = 10.2335 (7) Å	θ = 2.5–23.9°
*c* = 10.5784 (10) Å	µ = 0.81 mm^−^^1^
α = 104.559 (7)°	*T* = 293 K
β = 98.728 (7)°	Block, black
γ = 102.573 (7)°	0.25 × 0.12 × 0.11 mm
*V* = 708.01 (10) Å^3^	

### Data collection

**Table:**

Bruker APEXII CCD area-detector diffractometer	2878 independent reflections
Radiation source: fine-focus sealed tube	2395 reflections with *I* > 2σ(*I*)
graphite	*R*_int_ = 0.028
φ and ω scans	θ_max_ = 26.4°, θ_min_ = 3.2°
Absorption correction: multi-scan (*SADABS*; Sheldrick, 2003)	*h* = −8→8
*T*_min_ = 0.824, *T*_max_ = 0.916	*k* = −12→12
7213 measured reflections	*l* = −13→13

### Refinement

**Table:**

Refinement on *F*^2^	Primary atom site location: structure-invariant direct methods
Least-squares matrix: full	Secondary atom site location: difference Fourier map
*R*[*F*^2^ > 2σ(*F*^2^)] = 0.032	Hydrogen site location: inferred from neighbouring sites
*wR*(*F*^2^) = 0.076	H-atom parameters constrained
*S* = 1.01	*w* = 1/[σ^2^(*F*_o_^2^) + (0.0431*P*)^2^] where *P* = (*F*_o_^2^ + 2*F*_c_^2^)/3
2878 reflections	(Δ/σ)_max_ = 0.001
188 parameters	Δρ_max_ = 0.24 e Å^−^^3^
0 restraints	Δρ_min_ = −0.18 e Å^−^^3^

### Special details

**Table:**

Geometry. All e.s.d.'s (except the e.s.d. in the dihedral angle between two l.s. planes) are estimated using the full covariance matrix. The cell e.s.d.'s are taken into account individually in the estimation of e.s.d.'s in distances, angles and torsion angles; correlations between e.s.d.'s in cell parameters are only used when they are defined by crystal symmetry. An approximate (isotropic) treatment of cell e.s.d.'s is used for estimating e.s.d.'s involving l.s. planes.
Refinement. Refinement of *F*^2^ against ALL reflections. The weighted *R*-factor *wR* and goodness of fit *S* are based on *F*^2^, conventional *R*-factors *R* are based on *F*, with *F* set to zero for negative *F*^2^. The threshold expression of *F*^2^ > σ(*F*^2^) is used only for calculating *R*-factors(gt) *etc*. and is not relevant to the choice of reflections for refinement. *R*-factors based on *F*^2^ are statistically about twice as large as those based on *F*, and *R*- factors based on ALL data will be even larger.

### Fractional atomic coordinates and isotropic or equivalent isotropic displacement parameters (Å^2^)

**Table:**

	*x*	*y*	*z*	*U*_iso_*/*U*_eq_	
Cu1	0.5000	0.0000	0.0000	0.03685 (13)	
O	0.63653 (18)	−0.08439 (14)	0.10934 (13)	0.0465 (3)	
N	0.3572 (2)	0.06231 (15)	0.14161 (14)	0.0353 (3)	
C	0.2582 (3)	0.17043 (18)	0.14181 (17)	0.0348 (4)	
C1	0.3384 (3)	0.00334 (19)	0.23682 (18)	0.0374 (4)	
H1	0.2561	0.0326	0.2922	0.045*	
C2	0.5826 (3)	−0.13173 (18)	0.20525 (18)	0.0380 (4)	
C5	0.0594 (3)	0.14665 (19)	0.14435 (19)	0.0403 (4)	
H5	−0.0138	0.0584	0.1419	0.048*	
C6	−0.0300 (3)	0.2550 (2)	0.15063 (19)	0.0464 (5)	
H6	−0.1636	0.2380	0.1525	0.056*	
C7	0.3611 (3)	0.30083 (19)	0.13974 (19)	0.0443 (5)	
H7	0.4929	0.3167	0.1332	0.053*	
C8	0.3767 (3)	−0.16156 (19)	0.37004 (18)	0.0397 (4)	
C9	0.0726 (3)	0.3872 (2)	0.15411 (19)	0.0459 (5)	
C10	0.4283 (3)	−0.09993 (19)	0.26615 (18)	0.0366 (4)	
C12	0.2192 (3)	−0.1408 (2)	0.43269 (19)	0.0481 (5)	
H12	0.1454	−0.0824	0.4091	0.058*	
C14	0.4870 (3)	−0.24967 (19)	0.41041 (19)	0.0452 (5)	
C15	0.2694 (3)	0.4076 (2)	0.1473 (2)	0.0493 (5)	
H15	0.3417	0.4952	0.1478	0.059*	
C17	−0.0240 (4)	0.5069 (2)	0.1698 (3)	0.0680 (7)	
H17A	−0.1612	0.4714	0.1253	0.102*	
H17B	0.0411	0.5744	0.1308	0.102*	
H17C	−0.0132	0.5507	0.2631	0.102*	
C18	0.6458 (3)	−0.2737 (2)	0.3493 (2)	0.0530 (5)	
H18	0.7205	−0.3293	0.3779	0.064*	
C19	0.1725 (3)	−0.2047 (2)	0.5277 (2)	0.0595 (6)	
H19	0.0684	−0.1889	0.5678	0.071*	
C20	0.6920 (3)	−0.2190 (2)	0.2516 (2)	0.0493 (5)	
H20	0.7966	−0.2380	0.2136	0.059*	
C21	0.2798 (4)	−0.2931 (2)	0.5642 (2)	0.0651 (6)	
H21	0.2455	−0.3379	0.6270	0.078*	
C23	0.4339 (4)	−0.3138 (2)	0.5084 (2)	0.0602 (6)	
H23	0.5067	−0.3714	0.5350	0.072*	

### Atomic displacement parameters (Å^2^)

**Table:**

	*U*^11^	*U*^22^	*U*^33^	*U*^12^	*U*^13^	*U*^23^
Cu1	0.03127 (19)	0.0423 (2)	0.0432 (2)	0.01461 (14)	0.01515 (13)	0.01531 (15)
O	0.0397 (7)	0.0623 (9)	0.0530 (8)	0.0251 (7)	0.0218 (6)	0.0273 (7)
N	0.0314 (8)	0.0371 (8)	0.0410 (9)	0.0132 (7)	0.0112 (6)	0.0126 (7)
C	0.0350 (9)	0.0366 (10)	0.0338 (10)	0.0135 (8)	0.0105 (7)	0.0072 (8)
C1	0.0311 (9)	0.0421 (11)	0.0391 (11)	0.0110 (8)	0.0121 (8)	0.0083 (9)
C2	0.0334 (10)	0.0379 (10)	0.0413 (11)	0.0095 (8)	0.0072 (8)	0.0100 (9)
C5	0.0370 (10)	0.0388 (10)	0.0472 (11)	0.0122 (8)	0.0149 (8)	0.0110 (9)
C6	0.0369 (10)	0.0510 (12)	0.0538 (12)	0.0192 (9)	0.0159 (9)	0.0098 (10)
C7	0.0342 (10)	0.0436 (11)	0.0559 (13)	0.0101 (9)	0.0109 (8)	0.0157 (10)
C8	0.0415 (10)	0.0365 (10)	0.0368 (10)	0.0058 (8)	0.0065 (8)	0.0086 (8)
C9	0.0523 (12)	0.0435 (12)	0.0434 (11)	0.0232 (10)	0.0105 (9)	0.0067 (9)
C10	0.0349 (10)	0.0374 (10)	0.0373 (10)	0.0095 (8)	0.0082 (7)	0.0107 (8)
C12	0.0501 (12)	0.0532 (13)	0.0444 (12)	0.0134 (10)	0.0153 (9)	0.0179 (10)
C14	0.0548 (12)	0.0391 (11)	0.0396 (11)	0.0107 (9)	0.0063 (9)	0.0120 (9)
C15	0.0516 (12)	0.0363 (11)	0.0581 (13)	0.0090 (10)	0.0085 (10)	0.0148 (10)
C17	0.0777 (17)	0.0590 (14)	0.0781 (17)	0.0410 (13)	0.0208 (13)	0.0175 (13)
C18	0.0584 (13)	0.0500 (13)	0.0570 (14)	0.0249 (11)	0.0082 (10)	0.0210 (11)
C19	0.0625 (14)	0.0696 (15)	0.0463 (13)	0.0101 (12)	0.0208 (10)	0.0184 (12)
C20	0.0465 (12)	0.0539 (13)	0.0569 (13)	0.0255 (10)	0.0160 (9)	0.0195 (11)
C21	0.0892 (18)	0.0648 (15)	0.0485 (13)	0.0162 (14)	0.0222 (12)	0.0289 (12)
C23	0.0826 (17)	0.0534 (14)	0.0500 (13)	0.0206 (12)	0.0130 (12)	0.0233 (11)

### Geometric parameters (Å, °)

**Table:**

Cu1—O^i^	1.8837 (12)	C8—C14	1.417 (3)
Cu1—O	1.8837 (12)	C8—C10	1.452 (3)
Cu1—N	1.9848 (14)	C9—C15	1.382 (3)
Cu1—N^i^	1.9848 (14)	C9—C17	1.515 (2)
O—C2	1.302 (2)	C12—C19	1.373 (3)
N—C1	1.307 (2)	C12—H12	0.9300
N—C	1.434 (2)	C14—C23	1.414 (3)
C—C7	1.382 (2)	C14—C18	1.417 (3)
C—C5	1.384 (2)	C15—H15	0.9300
C1—C10	1.420 (2)	C17—H17A	0.9600
C1—H1	0.9300	C17—H17B	0.9600
C2—C10	1.408 (2)	C17—H17C	0.9600
C2—C20	1.431 (2)	C18—C20	1.343 (3)
C5—C6	1.385 (2)	C18—H18	0.9300
C5—H5	0.9300	C19—C21	1.390 (3)
C6—C9	1.378 (3)	C19—H19	0.9300
C6—H6	0.9300	C20—H20	0.9300
C7—C15	1.380 (2)	C21—C23	1.350 (3)
C7—H7	0.9300	C21—H21	0.9300
C8—C12	1.411 (3)	C23—H23	0.9300
			
O^i^—Cu1—O	180	C2—C10—C1	120.13 (16)
O^i^—Cu1—N	89.58 (5)	C2—C10—C8	119.57 (16)
O—Cu1—N	90.42 (5)	C1—C10—C8	119.94 (16)
O^i^—Cu1—N^i^	90.42 (5)	C19—C12—C8	121.51 (19)
O—Cu1—N^i^	89.58 (5)	C19—C12—H12	119.2
N—Cu1—N^i^	180	C8—C12—H12	119.2
C2—O—Cu1	128.62 (11)	C23—C14—C8	119.42 (19)
C1—N—C	115.44 (14)	C23—C14—C18	121.54 (18)
C1—N—Cu1	122.54 (12)	C8—C14—C18	119.03 (18)
C—N—Cu1	121.94 (11)	C7—C15—C9	121.53 (18)
C7—C—C5	118.92 (16)	C7—C15—H15	119.2
C7—C—N	120.13 (15)	C9—C15—H15	119.2
C5—C—N	120.95 (16)	C9—C17—H17A	109.5
N—C1—C10	127.97 (17)	C9—C17—H17B	109.5
N—C1—H1	116.0	H17A—C17—H17B	109.5
C10—C1—H1	116.0	C9—C17—H17C	109.5
O—C2—C10	124.11 (16)	H17A—C17—H17C	109.5
O—C2—C20	116.69 (16)	H17B—C17—H17C	109.5
C10—C2—C20	119.19 (17)	C20—C18—C14	122.24 (18)
C—C5—C6	119.60 (17)	C20—C18—H18	118.9
C—C5—H5	120.2	C14—C18—H18	118.9
C6—C5—H5	120.2	C12—C19—C21	120.4 (2)
C9—C6—C5	122.15 (17)	C12—C19—H19	119.8
C9—C6—H6	118.9	C21—C19—H19	119.8
C5—C6—H6	118.9	C18—C20—C2	120.91 (19)
C15—C7—C	120.38 (17)	C18—C20—H20	119.5
C15—C7—H7	119.8	C2—C20—H20	119.5
C—C7—H7	119.8	C23—C21—C19	120.0 (2)
C12—C8—C14	117.34 (17)	C23—C21—H21	120.0
C12—C8—C10	123.66 (17)	C19—C21—H21	120.0
C14—C8—C10	118.99 (17)	C21—C23—C14	121.4 (2)
C6—C9—C15	117.32 (17)	C21—C23—H23	119.3
C6—C9—C17	121.49 (18)	C14—C23—H23	119.3
C15—C9—C17	121.16 (19)		
			
O^i^—Cu1—O—C2	−71 (100)	C20—C2—C10—C8	−3.0 (3)
N—Cu1—O—C2	25.66 (16)	N—C1—C10—C2	11.9 (3)
N^i^—Cu1—O—C2	−154.34 (16)	N—C1—C10—C8	−174.99 (17)
O^i^—Cu1—N—C1	159.10 (14)	C12—C8—C10—C2	−177.19 (18)
O—Cu1—N—C1	−20.90 (14)	C14—C8—C10—C2	1.7 (3)
N^i^—Cu1—N—C1	−22 (100)	C12—C8—C10—C1	9.7 (3)
O^i^—Cu1—N—C	−17.40 (13)	C14—C8—C10—C1	−171.42 (16)
O—Cu1—N—C	162.60 (13)	C14—C8—C12—C19	−0.9 (3)
N^i^—Cu1—N—C	162 (100)	C10—C8—C12—C19	178.02 (18)
C1—N—C—C7	127.26 (18)	C12—C8—C14—C23	1.0 (3)
Cu1—N—C—C7	−56.0 (2)	C10—C8—C14—C23	−177.99 (17)
C1—N—C—C5	−52.6 (2)	C12—C8—C14—C18	179.69 (18)
Cu1—N—C—C5	124.13 (16)	C10—C8—C14—C18	0.7 (3)
C—N—C1—C10	−176.06 (16)	C—C7—C15—C9	−1.4 (3)
Cu1—N—C1—C10	7.2 (3)	C6—C9—C15—C7	−1.2 (3)
Cu1—O—C2—C10	−15.1 (3)	C17—C9—C15—C7	176.7 (2)
Cu1—O—C2—C20	166.16 (12)	C23—C14—C18—C20	176.8 (2)
C7—C—C5—C6	−2.8 (3)	C8—C14—C18—C20	−1.9 (3)
N—C—C5—C6	177.08 (17)	C8—C12—C19—C21	−0.3 (3)
C—C5—C6—C9	0.2 (3)	C14—C18—C20—C2	0.6 (3)
C5—C—C7—C15	3.4 (3)	O—C2—C20—C18	−179.34 (18)
N—C—C7—C15	−176.46 (17)	C10—C2—C20—C18	1.9 (3)
C5—C6—C9—C15	1.8 (3)	C12—C19—C21—C23	1.5 (4)
C5—C6—C9—C17	−176.09 (19)	C19—C21—C23—C14	−1.5 (4)
O—C2—C10—C1	−8.6 (3)	C8—C14—C23—C21	0.2 (3)
C20—C2—C10—C1	170.11 (16)	C18—C14—C23—C21	−178.5 (2)
O—C2—C10—C8	178.34 (16)		

Symmetry codes: (i) −*x*+1, −*y*, −*z*.
